# Artifact removal for unpaired thorax CBCT images using a feature fusion residual network and contextual loss

**DOI:** 10.1002/acm2.13968

**Published:** 2023-03-31

**Authors:** Wenqin Zhuang, Zheng Li, Haochen Liu, Hu Ying, Mo Yan

**Affiliations:** ^1^ College of Communications and Information Engineering Nanjing University of Posts and Telecommunications Nanjing China

**Keywords:** artifacts correction, contextual loss, FFRN, thorax CBCT

## Abstract

**Background and objective:**

Cone‐beam computed tomography (CBCT) has become a more and more active cutting‐edge topic in the international computed tomography (CT) research due to its advantages of fast scanning speed, high ray utilization rate and high precision. However, scatter artifacts affect the imaging performance of CBCT, which hinders its application seriously. Therefore, our study aimed to propose a novel algorithm for scatter artifacts suppression in thorax CBCT based on a feature fusion residual network (FFRN), where the contextual loss was introduced to adapt the unpaired datasets better.

**Methods:**

In the method we proposed, a FFRN with contextual loss was used to reduce CBCT artifacts in the region of chest. Unlike L1 or L2 loss, the contextual loss function makes input images which are not aligned strictly in space available, so we performed it on our unpaired datasets. The algorithm aims to reduce artifacts via studying the mapping between CBCT and CT images, where the CBCT images were set as the beginning while planning CT images as the end.

**Results:**

The proposed method effectively removes artifacts in thorax CBCT, including shadow artifacts and cup artifacts which can be collectively referred to as uneven grayscale artifacts, in the CBCT image, and perform well in preserving details and maintaining the original shape. In addition, the average PSNR number of our proposed method achieved 27.7, which was higher than the methods this paper referred which indicated the significance of our method.

**Conclusions:**

What is revealed by the results is that our method provides a highly effective, rapid, and robust solution for the removal of scatter artifacts in thorax CBCT images. Moreover, as is shown in Table 1, our method has demonstrated better artifact reduction capability than other methods.

## 1 INTRODUCTION

Cone beam computed tomography (CBCT) is widely used in the diagnosis of chest because of its advantages of a short scanning time and low X‐dose. Comparing to conventional fan‐beam computed tomography (CT), unfortunately, the quality of CBCT images degrades with the existence of truncated projections and X‐ray scattering. The problems lead to tremendous scatter artifacts, which severely impede the potential of CBCT in a variety of areas. As such, one of the primary difficulties encountered in CBCT is scattered radiation.^1^, ^2^, ^3^, ^4^

In recent years, researchers have conducted extensive research into scatter artifacts. Currently, the most popular methods for CBCT scatter correction via a digital image processing technology include analytical modeling methods, Monte Carlo (MC) simulation, CT‐prior‐based methods, histogram matching, and learning‐based methods.^5^ The analytical modeling method attempts to approximate the scattering distribution in projection data by assuming that the scattering signal is the convolution function of the main signal and the scattering kernel. An MC scattering estimation method based on GPU acceleration, variance reduction, and simulation has been proposed.^6^ This method uses less photon history and less sparse MC increment to accelerate the calculation time. However, a trade‐off between accuracy and simulation time occurs. CT‐prior‐based methods use DIR from CT to CBCT to obtain prior information.^7^ In histogram matching, the CBCT histogram of each slice is matched with the CT histogram by linear scaling to convert it to the correct degree.^8^ A learning‐based method, regression forest model, trained to correct CBCT images by extracting patient‐specific anatomical features from aligned CT and CBCT images.^9^ All these methods contribute positively but can still be improved. The last three methods require paired datasets, and the implementation is highly difficult.

Artifacts can also be improved by improving hardware, which reduces the number of scattered X‐rays arriving at the detector by considering the geometric conditions of X‐ray imaging to suppress artifact scattering.^10^, ^11^ Typically used methods include the air gap, collimator, filter, anti‐scattering grating, and modulator methods. However, based on the original CBCT system, hardware equipment for filtering and scattering will be added; hence, the operation of the CBCT system becomes more difficult, and the cost of the entire process is increased.^12^ Consequently, digital image processing is preferred.

In methods based on scatter estimation, the X‐ray spectral characteristics, object geometry and attenuation coefficients play a critical role. Additionally, the Klein‐Nishina formula and Spies model^13^ are essential for evaluating scatter artifacts. Inspired by the theories mentioned, Yao proposed a method to evaluate scatter artifacts approximately.^14^ Yang et al. have done relevant research, mainly assessing scattering caused by shadow areas additionally, and Stankovic et al. got scatter gram using a hybrid scatter evaluation method.^15^, ^16^ In addition, the results of level set and moving methods were also gratifying.

More researchers are using deep neural networks to manage scatter artifacts and achieve the desired results. Deep learning has become a general‐purpose solution for image‐to‐image transformation, and hand‐engineered mapping functions that require complicated formulations are not required anymore.^17^ Zhao determined the optimal parameters innovatively by introducing free parameters to convolution kernel of their model, producing an effect that scattering potential and the convolution kernel model can adapt to the evaluation of the scattering contour getting from image objects known previously perfectly.^18^ Baer et al. combined physical‐based scattering rectification methods and algorithms based on convolution operation, which achieve satisfying results.^19^ Yulun Zhang et al.^20^ proposed a residual dense network (RDN) to achieve super‐resolution images. In their experiment, they proposed a residual dense block (RDB) to extract abundant local features via dense connected convolutional layers. Their results showed that the RDB effectively suppressed artifacts in pictures. However, it did not function as intended when a CBCT image was used as an input. In 2021, Shipeng Xie and Tao Yang^21^ improved this method and proposed a residual skip dense block used in artifacts removal in sparse‐angle CT images. On this basis, this paper uses it in non‐aligned CBCT and CT images for further more improvement.

In 2018, Yang et al. proposed the use of deep residual convolutional neural networks to correct scattering artifact in thorax CBCT images,^22^ choosing the mean squared error (MSE) as the loss function of their neural network. The method structures a mapping between the corrected image and the original one using a framework which combines a deep learning network with a residual module. CBCT image blocks and CT image blocks were set as input images and labels, respectively. The experimental output data indicated that the method we proposed can reduce the artifacts in CBCT images effectively. However, a certain amount of distortion was observed at the pixel level between the input and the label, which did not correspond exactly.

To learn translation mappings in the absence of aligned paired images, J. Zhu et al.^23^ proposed cycle‐consistent adversarial networks (CycleGANs). S Kida et al. performed a visual enhancement on thorax CBCT images using CycleGANs. They put forward a comprehensive method based on CycleGANs with the aim of producing synthesized planning CT (SynPlanCT) from CBCT images. The method proposed merely depends on unpaired CBCT and planning CT (PlanCT) images to train our network. The quality of the comprehensive PlanCT images improved considerably, comparing with that of the original CBCT, furthermore it is able to preserve the anatomical structures of original CBCT well. The method could be applied directly to three‐dimensional CBCT images reconstruction, and it may facilitate the visualization of soft‐tissue details. Recently, Xiao Liang et al.^24^ used CycleGANs to generate synthesized CT (sCT) images from CBCT images for adaptive radiation therapy. Using their CycleGAN model, the Hounsfield unit (HU) accuracy of sCT images improved, compared to that of CBCT images, whereas the anatomy shown in sCT images remained the same as that in CBCT images. The HU values of the sCT images generated by CycleGANs are similar to those of the deformed planning CT (dpCT) images, whereas the anatomical accuracy achieved by CycleGANs is higher than that achieved by the DIR method.

**FIGURE 1:**
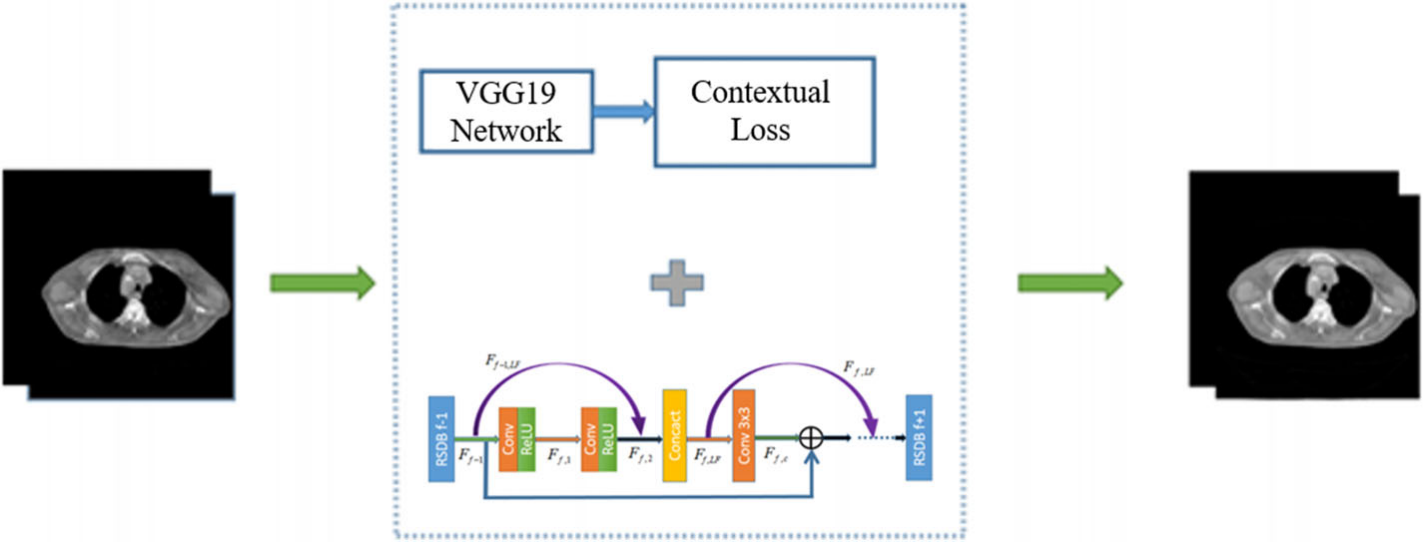
Flow chart of the proposed method.

In this study, to use non‐aligned paired images, we added contextual loss inspired by the method proposed by Roey^25^ to a feature fusion residual network (FFRN) to correct thorax CBCT artifacts. Contextual loss can solve the problem of pixel misalignment as it combines both context and semantics. Specifically, contextual loss compares regions with similar semantics, finds similar features, and then integrates the context of the entire image. This implies that contextual loss constrains the local features during the experimental process, allowing local deformation of data, which could make full use of misalignment data. The flow chart used in our method is shown in Figure 1.

The text arrangement of this paper is organized as follows. Firstly. in Section 2, we describe the method relevant to this topic concisely. Next, the methods proposed and the final results of our experiment are presented in Section 3. At last, conclusions and the outlook for future work are reported in Section 4.

## 2 MATERIALS AND METHODS

### 2.1 Data preprocessing

During the experiments, CBCT and CT images in the region of chest were set as input images and labels, respectively. We collected 2438 unpaired CBCT and CT 2D slices of chest from 18 different patients during IGRT with few motion artifacts. Since DICOM file not only stores patient's image information, but also contains patient's name, scanning device and other information, the latter is not needed for this task. Therefore, we extract the image information and store it in raw format, which makes the experimental data more concise. To adapt to our proposed network, the size of transformed images must be corrected to 512*512.

### 2.2 Contextual loss

The contextual loss function has excellent advantages in dealing with slight‐misaligned data, having a broad application prospect.^26^ To adapt to non‐aligned data, contextual loss measures the similarity between features by calculating the cosine distance between them; the smaller the cosine distance, the more similar the two images are. Therefore, the issue of assessing the similarity between two features is gradually converted into the challenge of minimizing the cosine similarity between feature graphs, effectively reducing the alignment requirements at the pixel level.

In our study, the local features were restricted by loss function, making it hopeful to operate on the areas which have similar semantics. To be specific, the operation contains the following steps: finding similar features in these similar semantic regions; forming a match between these features; achieving the similarity between the images.

Therefore, we can categorize this process into the following:

#### 2.2.1 Feature extraction

To calculate the similarity between features, we must extract features of target images first, and we selected the VGG19^27^ which has been trained on ImageNet^28^ previously as the extractor of the network we proposed. However, the VGG19 model pretrained was used to extract RGB images which have three channels, whereas our CT images were grayscale images. To adapt to this model, we used three identical slices as inputs. When processing digital images, feature extraction is mainly aimed at processing the features of the image, while RGB information is only a digital storage method of the original image. Since VGG19 network only pays attention to feature information of input images rather than RGB information, we can expand gray images into three channels if we want to apply the gray image to the RGB image scene, which can be realized by using three identical grayscale images as input. In addition, since these three channels have the same value, it will not affect the original features of the image.

**FIGURE 2:**
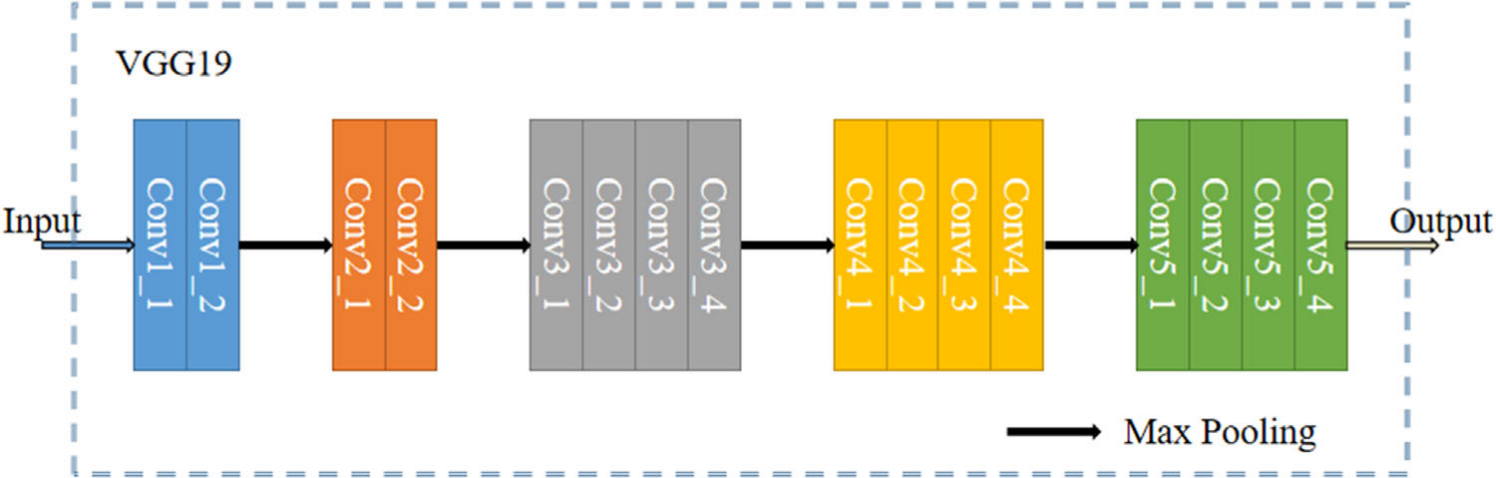
Network structure of VGG19.

Since the size of our data was 512*512, we chose VGG19 network which has deeper convolution structure so that deep texture features can be obtained easily. The VGG19 network was made up with 16 convolutional layers and three fully connected layers, max pooling was needed to increase receptive field. Additionally, we used the output of the network to get the loss function which we will introduce later, and VGG19 network can be briefly described as follow in Figure 2.

Compare the source thorax CBCT image *s* and target CT image *t* be the two images by the network; let $s_{i}$ and *t_j_* be the feature maps got from VGG19 net which is passed through by the source image *s* and target image *t*, respectively. So, source image *s* and the target image *t* can be seen as a set of feature map $s_{i}$ and $t_{j}$, respectively. Furthermore, we made a following rules that we assumed |S|=|T|=N, and when |S|≠|T|, N‐sampling was performed from a larger set.

#### 2.2.2 Similarity between features

Next, we need to calculate and evaluate the similarity between the two inputs. Relating to the cosine distance, contextual loss can measure how similar the two input images are. As follow, we would like to give a specific introduction to the contextual loss from mathematical methods, aiming to measure similarity between feature maps subsequently. Letting the value of $d_{ij}$ be the cosine distance of two target feature maps, its mathematical expression is defined as:

(1) $$d_{ij}=1-\frac{\left(s_{i}-\eta_{t}\right)\cdot \left(t_{j}-\eta_{t}\right)}{{\left\|s_{i}-\eta_{t}\right\|}_{2}{\left\|t_{j}-\eta_{t}\right\|}_{2}}$$

In the upper formula, $\eta_{t}=\frac{1}{N}\sum_{j}t_{j}$. We assumed that when $d_{ij}<<d_{ik},\forall k\neq j$, and some contexts of the feature maps $s_{i}$ and *t_j_* were similar. In order to get a simplified calculation, we rewrote formula (1) as follow:

(2) $${\tilde{d}}_{ij}=\frac{d_{ij}}{\min_{k}d_{ik}+\varepsilon }$$

Here, we define $\varepsilon =10^{-5}$. We convert distance into a measure of similarity by using an exponential operation, defined as:

(3) $$w_{ij}=\exp\left(\frac{1-{\tilde{d}}_{ij}}{h}\right)$$

In formula (3), *h* represents a bandwidth parameter, which is a positive number and is set as 0.5. Finally, using a scale‐invariant version of the normalized similarity, we defined the contextual similarity between the feature images as follow:

(4) $$CX_{ij}=\frac{w_{ij}}{\sum_{k}w_{ik}}$$

#### 2.2.3 Similarity between images

The calculation of contextual similarity between images can be defined mathematically as:

(5) $$CX\left(s,t\right)=CX\left(S,T\right)=\frac{1}{N}\sum_{j}\max_{i}CX_{ij}$$

Here, $CX_{ij}$ stands for the similarity between feature maps $s_{i}$ and $t_{j}$, which can be calculated by formula (4). However, a special case may occur in the calculation, which means the object of one of the image comparisons is itself. In this case, the value of feature similarity $CX_{xx}$ is 1, meaning that CX(S,S)=1.

#### 2.2.4 CX loss function

To sum up, the CX loss function is defined as:

(6) $$L_{CX}\left(s,t,l\right)=\log\frac{1}{CX(\phi^{l}\left(s\right),\phi^{l}\left(t\right))}$$

Here, ϕ is the symbolization of VGG19 network and *l* is the layer of network ϕ. $\phi^{l}\left(s\right)$ and $\phi^{l}\left(t\right)$ represent feature maps collected by *l* layer network ϕ from the image *s* and *t*.

### 2.3 The proposed loss function

We got the mapping from *s* to G(s) via training the network *G*, where an FFRN was selected and G(s) represented the output image of the model, and the source image *s* comprised CBCT image of the chest region with artifacts. The expression of the loss function is defined as:

(7) $$L\left(G\right)=\lambda \cdot L_{CX}\left(G\left(S\right),t,l_{t}\right)+L_{CX}\left(G\left(S\right),s,l_{s}\right)$$

Here, the value of $L_{CX}\left(G\left(S\right),t,l_{t}\right)$ indicates how similar the target CT images and generated images are, and the value of *L_CX_* (*G*(*S*),*s*,*l_s_*) is the symbolization of the similarity between input CBCT images and generated images. $l_{t}$ in formula (7) represents the style feature, whereas $l_{s}$ yields the content feature. In the upper formula, λ has a constant value and was set to 5, which dominates the ratio between two loss functions. It is worth noting that all the parameters were suitable for our experiments strictly through multiple experiments.

**FIGURE 3:**
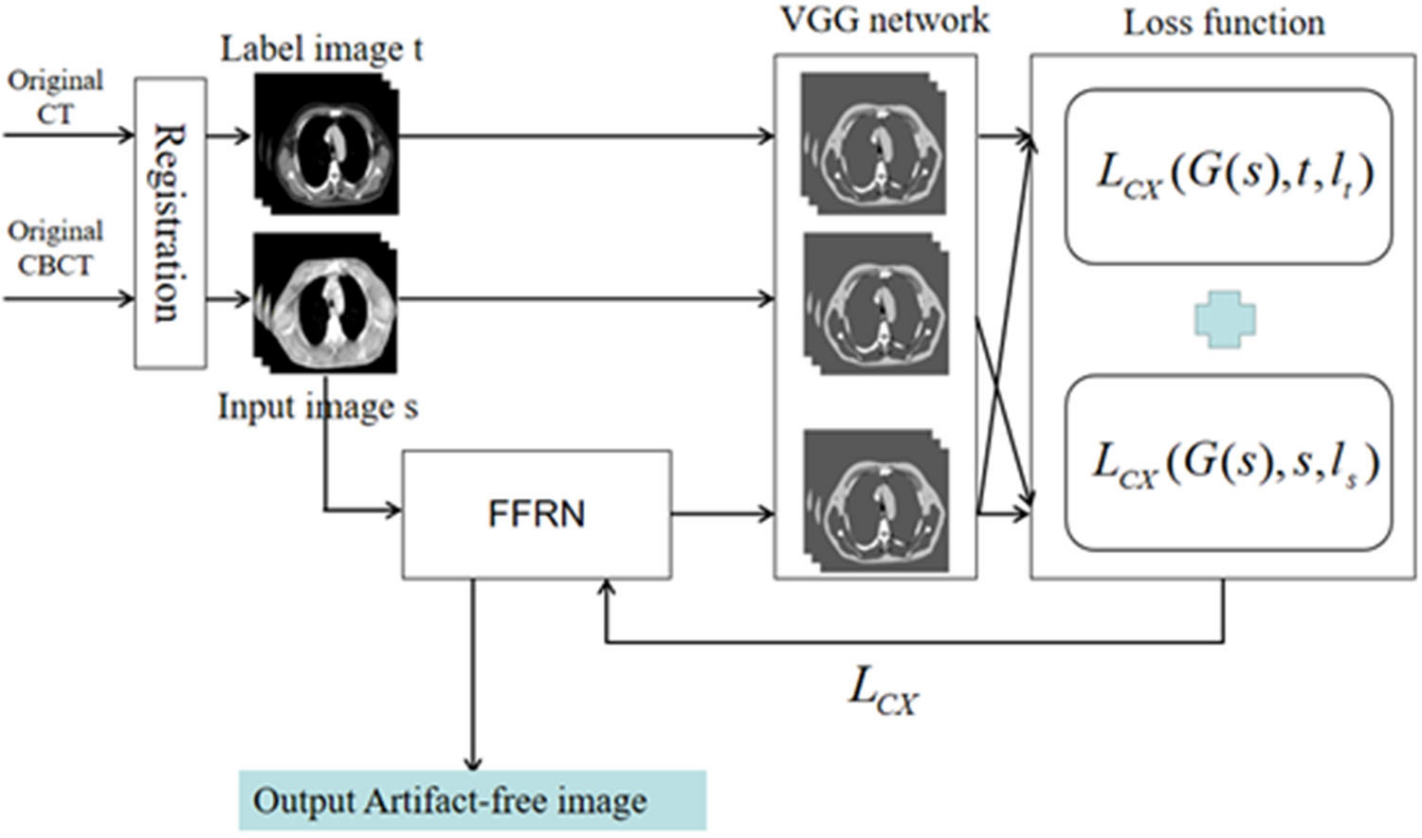
Experimental procession.

The detailed procession of our experiment can be briefly summarized shown in Figure 3.

### 2.4 Proposed network structure

#### 2.4.1 Residual block (RB)

**FIGURE 4:**
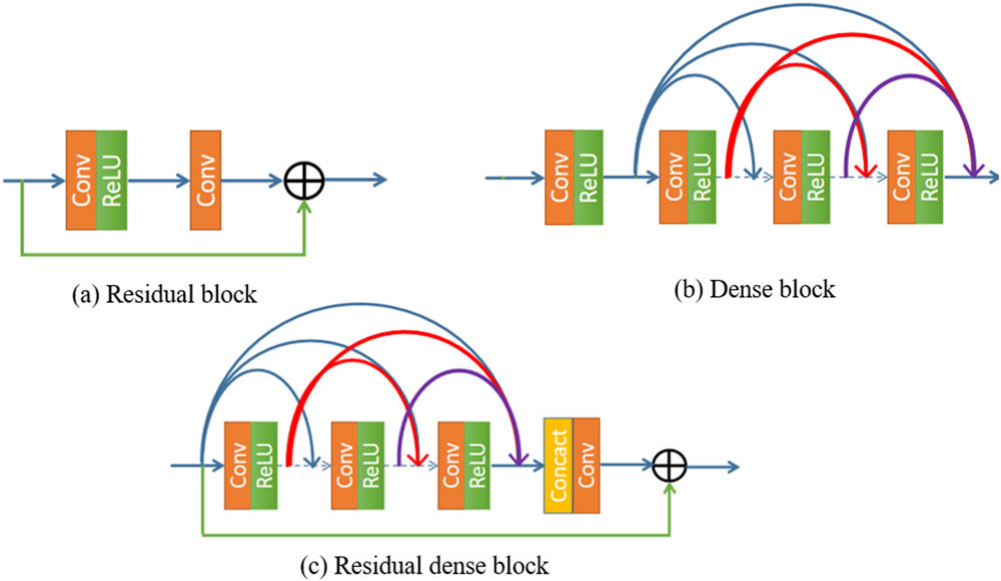
Common residual network building block.^20^

The structure of RB is shown in Figure 4, consisting of dense block, residual dense block and residual dense block, among which RDB can polish up the performance of the network further by abstracting multi‐level features. It takes advantage of whole layers through local dense connections, and afterward adaptively retains the cumulated features through local feature fusion (LFF). Combining both preface characteristics and deep features using globally residual learning, the RDB is able to acquire global dense features.

#### 2.4.2 Feature fusion residual network

**FIGURE 5:**
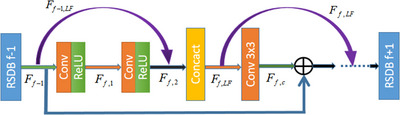
Residual skip dense block.

FFRN consists of RBs and residual skip dense blocks (RSDB). RSDB was proposed for artifacts reduction in sparse‐angle CT images, employing skip connections between blocks to merge local features and producing good results, as demonstrated in Figure 5.

Local feature fusion (LFF) is defined as:

(8) $$F_{f,LF}=C_{LFF}^{f}\left[F_{f-1,LF},F_{f,2}\right]$$

Here, $F_{f,LF}$ and $F_{f-1,LF}$ denote the (f)‐th and the (f‐1)‐th RSDB LFF result, respectively. $C_{LFF}^{f}$ represents the stack of concatenated feature maps, and $F_{f,2}$ symbols the first RSDB. $F_{f,2}$ can be expressed as:

(9) $$F_{f,2}=\delta \left\{W_{f,2}⊗\left[\delta \left(W_{f,1}⊗F_{f-1}+b_{1}\right)\right]+b_{2}\right\}$$

In formula (9), δ is the activation function (ReLU). $W_{f,1}$ and $W_{f,2}$ represent the weight of the first and second convolutional layers, respectively. *b*_1_ denotes the first bias, whereas *b*_2_ represents the second bias. $F_{f-1}$ symbols the result of the (f‐1)th RSDB output.

The stacked feature map can be expressed as follow:

(10) $$F_{f,LF}=C_{LFF}^{f}\left[F_{0,LF},F_{1,2},\text{…},F_{f,2}\right]$$

Here, $F_{0,LF}$ represents the feature map of the Conv layer in the model adaptive RB in front of the first RSDB.

**FIGURE 6:**
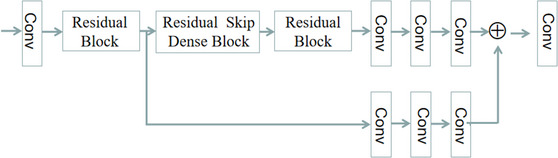
FFRN structure.

Figure 6 shows the FFRN structure used in our experiment. Since our network was able to adapt to the task of artifacts removal in sparse‐angle artifact CT images well, we changed the number of convolution layers to adapt our experiments on this basis.

In our network structure, the local feature fusion layer was used behind three convolution layers of the building block. The RSDB skips the connection of the local feature fusion layer, and the result of previous module feature fusion will be input to the latter module. Subsequently, overlay the features fused by local feature fusion layers, and integrate the feature information with residual information to shape a basic network structure.

## 3 RESULTS

To validate our experimental results, we compared our results with those obtained using the methods described in Ref.[26] and Ref.[29] Compared to the former method,^26^ our method was more effective in artifact correction and image detail preservation during artifact removal. Furthermore, we compared our method with that discussed in Ref.,[29] as the U‐net is known for preserving texture details in images, creating near‐real image manifolds, and producing visually convincing solutions. The results show that our method yielded better image reconstruction quality. Further details will be provided in the experimental results, and we will describe the improvement process used to enhance the network performance.

### 3.1 Dataset

Owing to the necessity of the dataset used for convolutional networks training, we have to access a great number of datasets to ensure that the model can be trained sufficiently to represent the full sample spaces. We used chest data from patients to train the proposed model for clinical application. With the cooperation of hospital, we collected 2438 unpaired thorax CBCT and CT 2D slices of chest from 18 different patients during IGRT with few motion artifacts. The unpaired images were randomly selected from patients IGRT in the same inpatient region. Also, we collected 200 paired CBCT and CT 2D slices for testing. Specially, for the patients, a CT scan of the treatment area should be undergone before the start of IGRT. To minimize radiation exposure to the patients, doctors often recommend that patients use CBCT scans instead of CT scans in subsequent radiotherapy.

During training the network, the size of each images was resized to 512 × 512, and the format of per images were changed to raw to suit the network training. Our dataset contains unpaired CBCT and CT images, and we use CBCT images with severe artifacts as the input of networks. Meanwhile, CT images with high imaging quality from the same patient are set as the corresponding labels of CBCT.

### 3.2 Network training

To be able to ameliorate the quality of thorax CBCT images with massive severe artifacts, we must acquire a rigorous mapping between CBCT and CT images by means of training the network, while the CBCT images were set as the beginning and planning CT images as the end. Firstly, thorax CBCT images were inputted into FFRN module to get preliminary processed images, and then, the input, generated and label images were input to the VGG19 for feature extraction.

**FIGURE 7:**
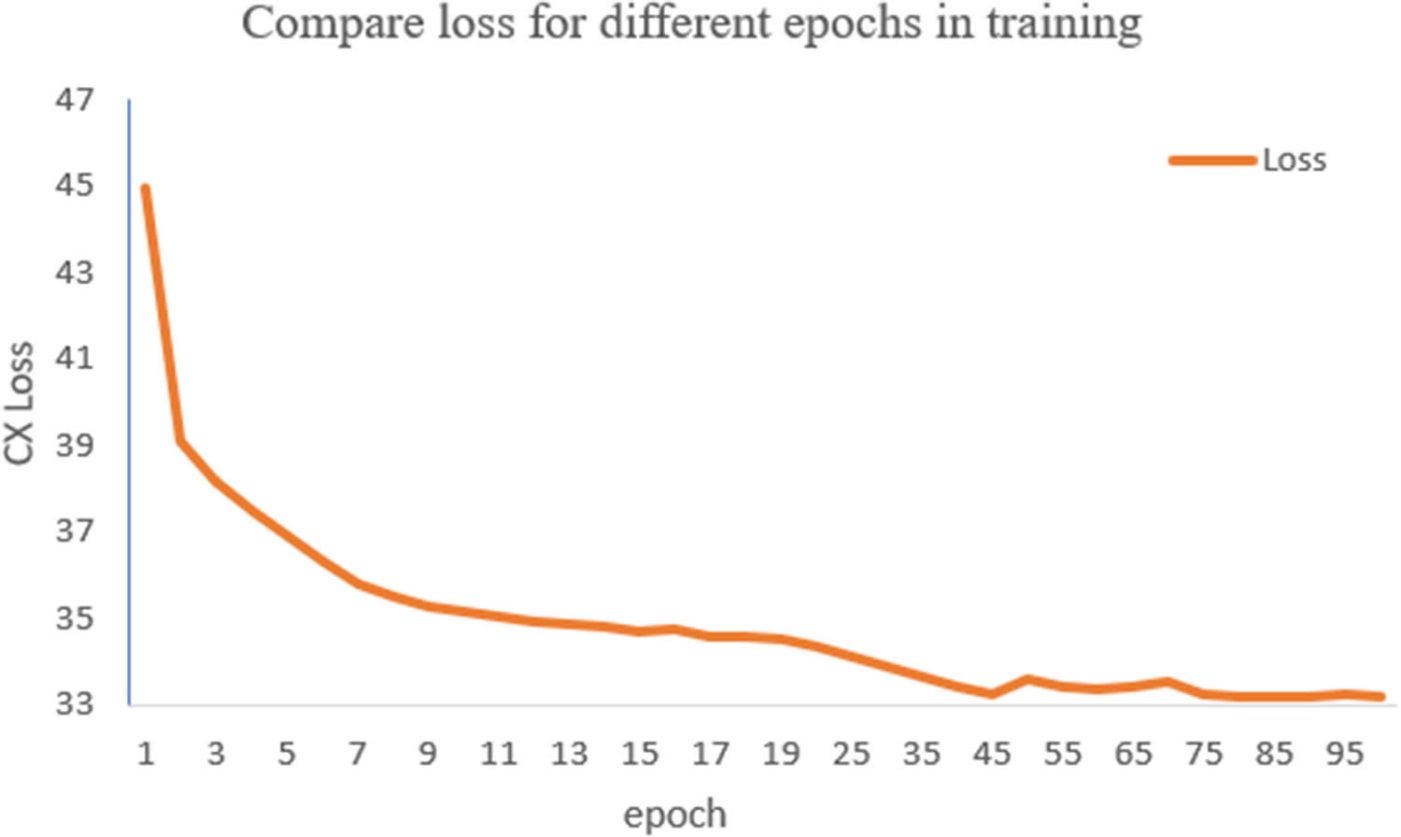
The loss change in different epochs.

During the training phase of the network, we can compute the contextual loss on the basis of the characteristics of relevant convolutional layers as well as the difference in pixel values between CBCT and CT images. As the value of contextual loss changes, that of the reconstructed CT images would also update. During the experiment, we used iterative optimization to correct the thorax CBCT images. Since the general training effect of the model can be reflected by the changes of value and convergence of loss function objectively, we should compare the effects of diverse network parameters on the artifacts correction results. The relationship between epoch and contextual loss value in our experiment is shown in Figure 7. According to the results of artifacts removal, the ratios of contextual loss function, network parameters, and feature sample size were adjusted whenever necessary, and the network was trained repeatedly until it is optimal for the artifact removal task.

In our experiment, we used the TensorFlow library on a GeForce GTX 1080 Ti processor in Python environment. To optimize our network, Adam optimizers were selected and ReLU activation function was used in this study. The input and label images were set to 512 × 512 as well as output images. The value of "epoch" was set to 100 and "learning rate" was 1e−4 when we trained the network. To achieve an accurate convergence, we set the "step size" to 2. According to the traditional method employed in deep learning field,^30^ every convolution layer uses a kernel with the size of 3 × 3.

The peak signal‐to‐noise ratio (PSNR) was used as the evaluation function for our model training. The input of the PSNR was (x,G(x)), where *x* represents real target image while G(x) represents generated predicted image. The expression of the PSNR function is as follows:

(11) $$PSNR=10\cdot \log_{10}\left(\frac{{\left(2^{n}-1\right)}^{2}}{MSE}\right)$$

In the upper function, *n* represents the number of per pixel bites. We set it to nine so that the pixel gray scale was 512. The unit of PSNR is DB, and the PSNR value is proportional to the image quality, while the value of PSNR is inversely proportional to the distortion. Mean square error (MSE) represents the mean square error between target image *x* and predicted image G(x). The MSE function can be expressed as follow:

(12) $$MSE=\frac{1}{H\times W}\sum_{i=1}^{H}\sum_{j=1}^{W}{\left(x\left(i,j\right)-G\left(x\right)\left(i,j\right)\right)}^{2}$$

In function (12), H and W are the height and width of images, respectively. *i*&*j* represent image pixel value at (i,j) position.

### 3.3 Experiment results

**FIGURE 8:**
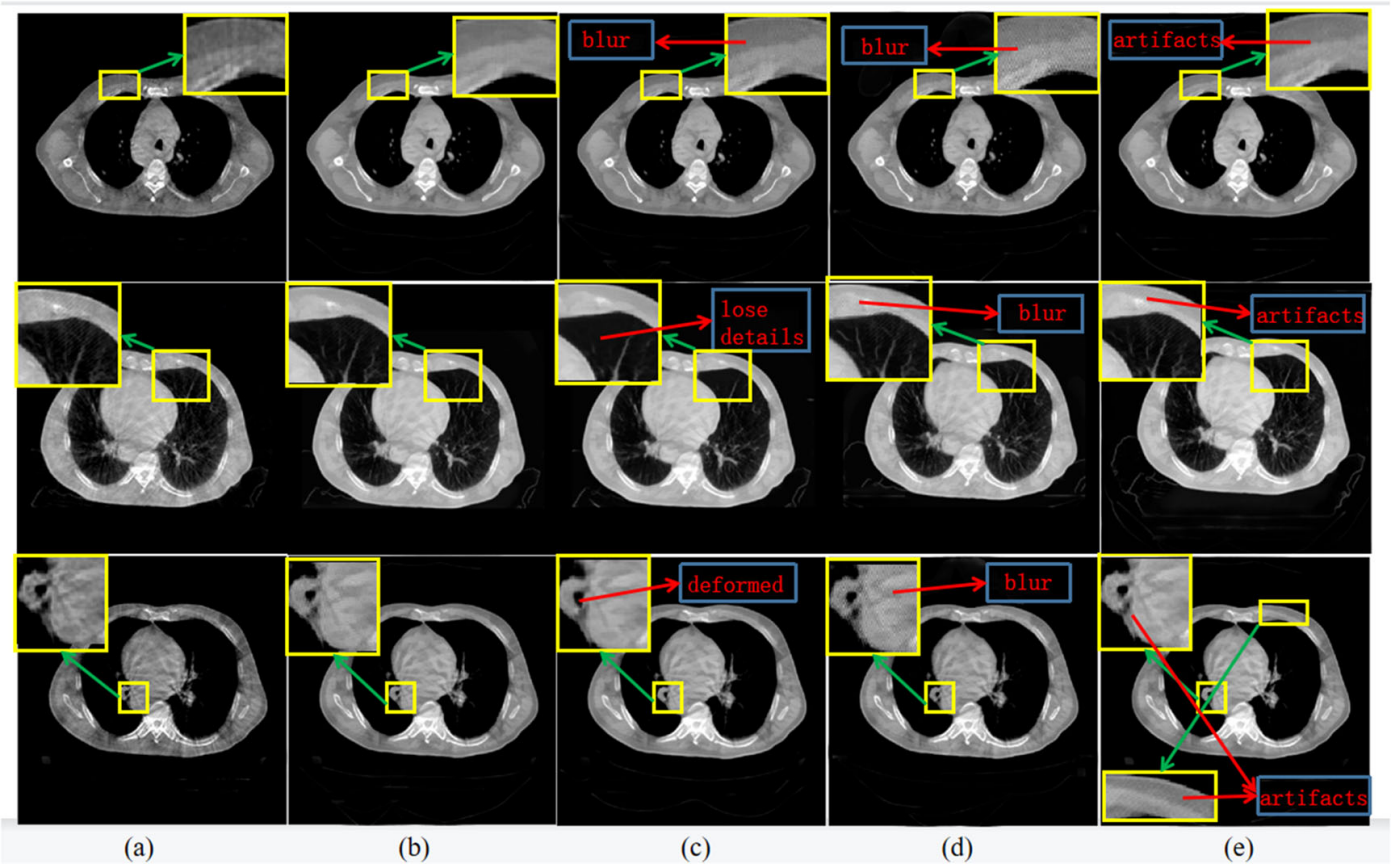
Artifact removal results obtained with chest data. (a) CBCT, (b) proposed correction, (c) method in Ref.,[26] (d) method in Ref.,[29] (e) method in Ref.[31] Row two display window [0,1528]. Other display window [498,1528].

By analyzing the results presented in Figure 8, we can get that the method we proposed can achieve the goal of removing scattering artifacts in thorax CBCT slices effectively. To get a better visual representation, we used Adjust contrast tool in MATLAB to set an appropriate HU value window, and the specific HU window values will be provided later. In Figure 8, the first column represents the original CBCT slices, which are heavily contaminated with many streak artifacts so that the diseased region could not be observed thoroughly, and some details corrupted might influence professionals’ judgment. The second column is the correction results by our proposed method. As the results show below, basically, all the stripe artifacts are suppressed, and the detail texture of the source image is not destroyed. The third column is the results obtained using the method in.^26^ In the first line of Figure 8, the marked region in source image is a bone area, which is contaminated by artifacts. The result obtained in method^26^ did not remove artifacts entirely since it almost eliminates all the textures at the nodule, while our method makes the branches at the nodule appear more clearly. The same condition appears in the marked area of the third line again, comparing with our results, the method^26^ has lost some details of the original image, which may cause doctors to misjudge in clinical diagnosis. Besides this, the esophagus area in the third column has obvious deformation. The forth column is the results obtained in method,^29^ which shows many segmented image blocks. The reason for this condition is that the U‐Net architecture is arranged down samples operation for four times, amounting to 16 times, and, similarly, in its decoder part, up‐samples are performed for four times symmetrically, aiming to restore the high‐level feature map gotten by encoder part to the input‐size resolution, so this method is more suitable for image segmentation tasks rather than our task. Finally, the fifth column is the results obtained in method.^31^ The images below present a problem that the artifacts are not removed entirely.

It is generally accepted that quantitative analysis can help us evaluate and compare the effects of different artifact removal methods more effectively. Here, we presented the results obtained from chest region in the following Table 1, including average PSNR and the values of average CT numbers. Each value was obtained by the experiment on the test datasets and taking the average, and we used the analysis measurement function in ImageJ to calculate the average CT number. The average CT numbers of bone and skin we calculated in test datasets were compared with those CT numbers coming from the initial CT slices. By comparing the discrepancy in CT numbers obtained in various methods, we can identify the better one. As the Table 1 shows below, our method got the highest average PSNR value, up to 27.7, which indicates that the method we proposed achieved the best performance among the methods involved. In addition, compared with the methods in Ref.,[26] the numbers of CT slices which has been corrected by our method in this paper were closer for the regions of interest comparing to these of initial CT images which indicates that the well‐trained FFRN can reduce the artifacts more effectively.

**TABLE 1:** Quantitative analysis of different methods.

Evaluation indicators		CBCT	CT	FFRN+CX	CNN+CX	U‐net+CX	GAN+Per
Mean CT numbers (Hu)	Bone marrow	221.4	231.8	**227.6**	226.8	223.6	223.4
	Skin	−186.6	−140.4	**−147.0**	−147.4	−180.2	−160.9
Average PSNR		26.3	/	**27.7**	26.7	24.1	24.4
Average SSIM		0.88	/	**0.89**	0.88	0.80	0.80
MAE		39.0	/	**33.4**	34.6	36.8	35.7

The bold values in the table are the results of our proposed method.

**FIGURE 9:**
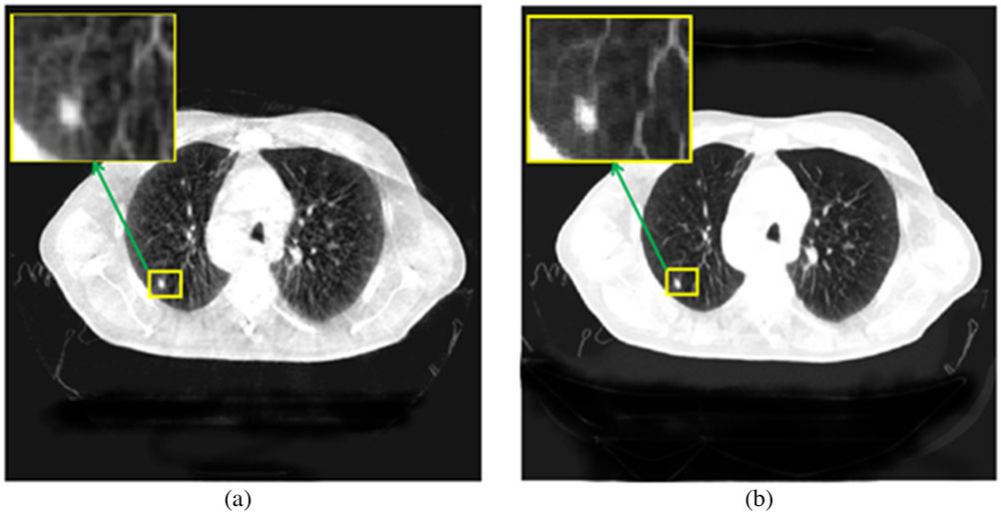
Spiculation tumor. Display window [−100,1063]. (a) The original CBCT image; (b) the correction result of our proposed method.

In addition, we chose two images with pathological area to analyze the efficiency of our proposed method. Lung tumor has diverse forms including lobulation, spiculation, nodule sign, vacuole sign, cavity sign, honeycomb sign and so on. Figure 9 is spiculation, which refers to 3 ∼ 15 burr shadows protruding from the edge of the nodule, and is caused by interstitial hemorrhage, exudation and fibrosis in the lung. The left image of Figure 9 is the original CBCT image, which is visually blurred, and the right image is more limpid so that it can be easy to attract the attention of clinicians and improve the accuracy of judgment.

**FIGURE 10:**
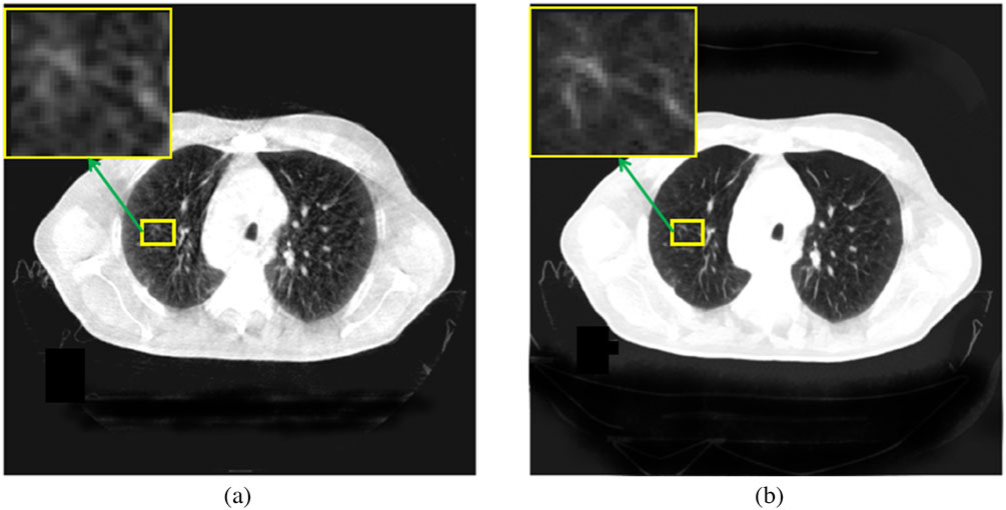
Vacuole sign tumor. Display window [−100,1063]. (a) The original CBCT image; (b) the correction result of our proposed method.

As shown in Figure 10, vacuolar tumors are characterized by showing 1−2 mm punctate translucent shadows on CT images, mostly multiple, whose pathological basis is mainly alveoli, dilated and twisted bronchioles. The results of our proposed method are on the right, which better shows the location of the lesion. In addition, our approach removes the illusion effectively that there appear to be three vacuole signs on the left, but in fact there are two. Finally, as this vacuole can cause adenocarcinoma or alveolar cancer, it needs to receive adequate attention in diagnosis, and the image processed by our method can better assist doctors in making judgments.

What we can get from the result is that, the performance of our proposed method outstands the control group methods both in terms of artifacts removal and the complexity of computation, which could help break the limitation of clinical applications. In addition, Table 2 compares the time of test between the model we proposed and others^26^ with the same computational power, indicating that the operation efficiency of our method has been improved greatly.

**TABLE 2:** A comparison between the testing time of the proposed network and method.^26^

Method	Proposed method	Method^26^
Average test time/chest image	0.072s	0.229s
Average test time/head image	0.074s	0.213s

## 4 DISCUSSION AND CONCLUSION

Because of its low X‐ray dose, CBCT is less harmful to the human body than CT, so it has been used more and more widely in the world, but the existence of severe artifacts in CBCT images affects its imaging quality and the clinical diagnosis of doctors. Therefore, in this paper, we intend to use thorax CBCT and CT images of the same patient in artifact correction task, so that CBCT images contaminated by severe artifacts can achieve high quality like high‐resolution CT images. However, the problem is that CBCT and CT images do not have same temporal resolutions because of the difference of their imaging principle, which means that strictly aligned CBCT and CT images cannot be accessed easily, so non‐aligned CBCT&CT image pairs in the region of chest were used to solve this problem.

Based on cosine similarity, contextual loss was introduced to feature layer abstracted by VGG19 network innovatively, which can handle non‐aligned images well. Since Contextual loss is based on semantics while feature similarity decides image similarity evaluation results, instead of distance, it can withstand slight data movements well and the problem of mismatched data be solved.

Moreover, in our scatter artifact correction task, we selected FFRN as our feedforward network, which significantly improved the quality of images generated by the actual CBCT system, reducing both the area and intensity of scattering artifacts. Furthermore, the generalization of this method allows it to be applied to other anatomies and organs, though our test dataset consists of chest CBCT images. The superiority of our method is evident, as anatomical features in thorax CBCT were extracted and used as training data, while still preserving pathological information details, such as textures present in the internal contour of chest regions. As the results showed, our method performed excellently in the task of removing artifacts from thorax CBCT images.

If more complicated generation networks were used, the effect of our method could be further boosted. Future researches will focus on inquiring into related technologies further as well as improving our experimental results.

## AUTHOR CONTRIBUTIONS

Wenqin Zhuang (Corresponding Author & First Author): Project administration; Conceptualization; Writing—Original Draft; Software. Zheng Li: Writing—Review & Editing; Visualization; Software. Haochen Liu: Formal analysis; Data Curation; Validation; Software. Hu Ying: Formal analysis; Data Curation; Validation; Software. Mo Yan: Conceptualization; Software.

## CONFLICT OF INTEREST STATEMENT

Authors have no conflict of interest to declare.

## Data Availability

The data that support the findings of this study are available on request from the corresponding author. The data are not publicly available due to privacy or ethical restrictions.
